# Identification of Biomarkers to Construct a Competing Endogenous RNA Network and Establishment of a Genomic-Clinicopathologic Nomogram to Predict Survival for Children with Rhabdoid Tumors of the Kidney

**DOI:** 10.1155/2020/5843874

**Published:** 2020-08-26

**Authors:** Xiaoqing Wang, Xiangyu Wu, Tianyou Li, Mingyu Cui, Lichao Zhu, Gang Wang, Feng Guo

**Affiliations:** Department of Pediatric Surgery, Shandong Provincial Hospital Affiliated to Shandong First Medical University, Jinan 250021, China

## Abstract

Rhabdoid tumor of the kidney (RTK) is a rare and severely malignant tumor occurring in infancy and early childhood, with the overall outcomes remain poor. Neither gene regulatory networks nor biomarkers to predict the prognostic outcomes have been elucidated in RTK. In this study, RNA sequencing data were obtained to identify differentially expressed messenger RNAs (mRNAs), long noncoding RNAs (lncRNAs), and microRNAs (miRNAs) between RTK samples and normal samples. A total of 4217 mRNAs, 284 lncRNAs, and 286 miRNAs were screened out. Of those, 103 mRNAs, 80 lncRNAs, and 45 miRNAs were identified for a competing endogenous RNA (ceRNA) regulatory network, in which three significant modules were identified. A protein-protein interaction (PPI) network was constructed, and the hub-gene cluster consisted of four core genes (EXOSC2, PAK1IP1, WDR43, and POLR1D) was selected. Gene ontology (GO) and Kyoto Encyclopedia of Genes and Genomes (KEGG) pathway enrichment analyses were also performed to analyze the functional characteristics of differentially expressed mRNAs. Subsequently, among 211 mRNAs, 8 lncRNAs, and 12 miRNAs associated with overall survival (OS) obtained by univariate Cox analysis, 5 mRNAs, 7 lncRNAs, and 7 miRNAs were identified and the risk score formulas were constructed correspondingly using the least absolute shrinkage and selection operator (LASSO) Cox regression model analysis. The log-rank tests and Kaplan-Meier analyses were performed to confirm the predictive value of the risk scores for OS in RTK patients. A genomic-clinicopathologic nomogram integrating the stage and risk scores based on RNAs was established and demonstrated high predictive accuracy and clinical value, which was validated through calibration curves, time-dependent receiver operating characteristic (ROC) curve analyses, and decision curve analysis (DCA). In conclusion, this study not only provided potential insights into the mechanisms underlying RTK, but also presented a practicable tool for predicting the prognosis in children with RTK.

## 1. Introduction

As rare and extremely aggressive malignancies, rhabdoid tumors primarily affect infants and young children. These tumors predominantly arise in the kidney and the central nervous system [1, 2]. Rhabdoid tumor of the kidney (RTK) generally metastasizes to the brain and lung, and patients with RTK continue to have a poor prognosis [1, 3, 4]. The loss of function of the SMARCB1 (INI1/SNF5/BAF47) gene is the common genetic abnormality in rhabdoid tumors, regardless of the anatomic origin [5]. However, there are few reports on the development of biomarkers to predict the prognostic outcomes in children with RTK.

In recent years, advanced RNA sequencing analysis gained a lot of attention and revealed the complexity of the human genome [6]. Under such circumstances, competing endogenous RNA (ceRNA) hypothesis has stated that long noncoding RNAs (lncRNAs) can act as microRNAs (miRNAs) sponges and inhibit miRNAs functions by sharing miRNA response elements, thereby indirectly regulating messenger RNAs (mRNAs) expression levels [7, 8]. Some previous studies have explored the ceRNA regulatory network associated with tumor progression [9–11]. However, the specific ceRNA regulatory network remains unelucidated in RTK.

In the present study, RNA sequencing data were used to identify differentially expressed mRNAs, lncRNAs, and miRNAs between RTK samples and normal samples. Subsequently, a series of analyses, including the ceRNA network construction, protein-protein interaction (PPI) analyses, and functional enrichment analyses, were performed. Furthermore, risk scores based on mRNAs, lncRNAs, and miRNAs to predict the outcomes in patients with RTK were calculated, and a genomic-clinicopathologic nomogram, integrating the risk scores and traditional clinicopathological factors, was developed and validated. All of these might not only provide insights into the molecular mechanisms that participate in the progression and tumorigenesis of RTK but also provide an efficient method based on biomarkers to predict the outcomes in children with RTK.

## 2. Materials and Methods

### 2.1. Study Population and RNA Sequencing Data Processing

**Figure 1:**
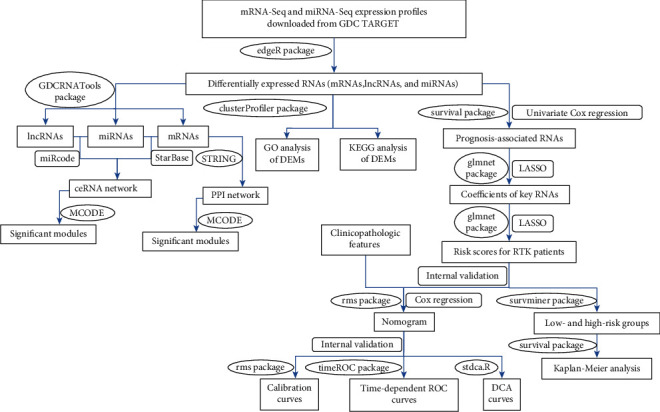
The flow chart of the analysis procedure. GDC: Genomic Data Commons Data Portal; TARGET: Therapeutically Applicable Research to Generate Effective Treatments; ceRNA: competing endogenous RNA; PPI: protein-protein interaction; GO: Gene Ontology; KEGG: Kyoto Encyclopedia of Genes and Genomes; DEMs: differentially expressed mRNAs; LASSO: the least absolute shrinkage and selection operator; RTK: rhabdoid tumor of the kidney; ROC: receiver operating characteristic; DCA: decision curve analysis.

Expression profiles (mRNA-Seq and miRNA-Seq), clinical characteristics, and survival data were downloaded for subsequent analysis from the National Cancer Institute (NCI) Genomic Data Commons (GDC) Data Portal (https://portal.gdc.cancer.gov/repository), Therapeutically Applicable Research to Generate Effective Treatments (TARGET) Program in April 2020, through structured queries [12]. mRNAs and lncRNAs expression data were acquired from 6 normal samples and 57 RTK samples, and miRNAs expression data were acquired from 6 normal samples and 58 RTK samples. ENSEMBL (htps://http://www.ensembl.org/) was used to annotate mRNAs and lncRNAs [13], and miRBase 21 (http://http://www.mirbase.org/blog/2014/06/mirbase-21-finally-arrives/) was used to annotate mature miRNAs with arms features through the R software (version 3.6.2) [14–19]. No ethical approval was required for the study as all patient data were acquired from the GDC Data Portal. A flow chart of the analysis procedure is shown in Figure 1.

### 2.2. Identification of DEMs, DELs, and DEMis

At first, RNAs (mRNAs, lncRNAs, and miRNAs) that did not have a worthwhile number of reads in any samples were filtered out. To identify the differentially expressed mRNAs (DEMs), lncRNAs (DELs), and miRNAs (DEMis) between RTK samples and normal samples, the “edgeR” package (version 3.28.1) was used to analyze high-throughput sequencing data on differentially expressed RNAs [20, 21]. The screening conditions for RNAs (mRNAs, lncRNAs, and miRNAs) differential expression were ∣log_2_ fold change | >1, and a false discovery rate (FDR) or adjusted *P* value < 0.05. These differentially expressed RNAs were further subjected to the ceRNA network construction, gene enrichment analysis, and RNA-based prognostic model construction combined with clinicopathologic features.

### 2.3. Construction of ceRNA Network

Basing on DEMs, DELs, and DEMis, a ceRNA network was built through the “GDCRNATools” package (version 3.28.1) [22]. The major criteria for building ceRNA networks included the following: (1) The mRNAs and lncRNAs must share a significant number of miRNAs. (2) The expression of mRNAs and lncRNAs should be related positively. (3) miRNAs should play similar roles in regulating the expression of mRNAs and lncRNAs [22]. Following the pipelines of GDCRNATools, miRcode was chosen to collect predicted and experimentally validated miRNA-lncRNA interactions [23], and StarBase v2.0 was used to predict miRNA-mRNA interactions [24]. Also, the *P* value of both the hypergeometric test and Pearson correlation analysis <0.01 was considered statistically significant. Visualization of the ceRNA network was performed by Cytoscape software (version 3.7.1) [25], and the subnetwork was generated using the Molecular Complex Detection (MCODE) plug-in Cytoscape with the default criteria (degree cutoff = 2, node score cutoff = 0.2, Max depth = 100, and *k* score = 2) [26].

### 2.4. PPI Network Construction

The construction of PPI network based on the DEMs involved in the ceRNA network was performed through the Search Tool for the Retrieval of Interacting Genes (STRING; http://string-db.org) online database [27]. Required interaction score > 0.4 was considered statistically significant. Subsequently, the data from STRING were downloaded to model the PPI network through Cytoscape software. MCODE plug-in Cytoscape with the default criteria was adopted to identify densely connected regions in the PPI network.

### 2.5. GO and KEGG Pathway Analyses

Gene Ontology (GO) functional enrichment analyses including biological process (BP), cellular component (CC), and molecular function (MF) [28, 29] and Kyoto Encyclopedia of Genes and Genomes (KEGG) pathway enrichment analyses of the DEMs [30–32] were performed through the “clusterProfiler” R package (v3.6.0) [33]. The *P* value < 0.05 was considered statistically significant.

### 2.6. Construction of Risk Scores Based on RNAs for Survival Analyses

Due to the lack of important clinical information such as the survival time of some patients, clinical data of 50 RTK patients were finally collected. In this study, the clinical features include age at diagnosis, gender, cooperative group protocol, and stage (Table 1). The normalization of the high-throughput sequencing raw data had already been carried out by “edgeR”, and the normalized expression data were converted to log_2_counts per million (log_2_CPM) values using the “cpm” function in edgeR. The “survival” package in R was used to identify the prognosis-associated RNAs (mRNAs, lncRNAs, and miRNAs) by univariate Cox regression analysis, and *P* < 0.05 was used as the cutoff criterion [34]. Subsequently, through the “glmnet” package (version 3.0-2) in R, the least absolute shrinkage and selection operator (LASSO) method was adopted to identify the key RNAs from RNAs which were significant in univariate Cox analysis [35, 36]. Ten-time cross-validations were utilized to detect the best penalty parameter lambda. Then, the risk scores were calculated based on the formulas generated through the LASSO Cox regression model, respectively. The optimal cutoff values for risk scores were generated through the “surv_cutpoint” function in the “survminer” R package (version 0.4.6). Based on cutoff values, RTK patients in the data set were divided into low- and high-risk groups correspondingly. Differences in overall survival (OS) between the low- and high-risk groups were compared via the log-rank test and Kaplan-Meier analysis. The survival curves were also constructed through the “survival” package. A *P* < 0.05 denoted statistical significance.

### 2.7. Establishment and Validation of a Genomic-Clinicopathologic Nomogram

The Cox regression analysis was performed to determine whether the risk scores based on RNAs and the clinicopathologic features could be predictors associated with OS for RTK patients. Subsequently, based on the results of Cox regression analysis, a predictive genomic-clinicopathologic nomogram was generated to predict 1-, 3-, and 5-year OS through the “rms” package (version 5.1-4) in R. Furthermore, calibration curves were achieved to visualize the consistency between the actual probability of OS and the nomogram-predicted probability of OS. Moreover, time-dependent receiver operating characteristic (ROC) curve analyses were performed to assess the accuracy by calculating the area under the curve (AUC) through the “timeROC” package (version 0.4) in R [37]. Additionally, decision curve analyses (DCA) were conducted to examine the clinical value via “stdca.R” statistical code in R [38].

## 3. Results

### 3.1. Identification of DEMs, DELs, and DEMis

**Figure 2:**
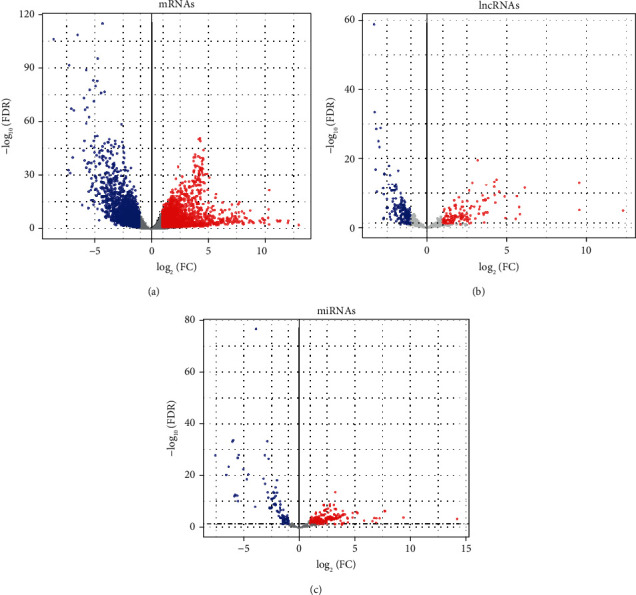
Volcano plots of differentially expressed mRNAs (a), lncRNAs (b), and miRNAs (c). The red dot represents upregulated and the blue dot represents downregulated. FDR: false discovery rate; FC: fold change.

A total of 4217 mRNAs, 284 lncRNAs, and 286 miRNAs were considered to be differentially expressed in the present study. Strikingly, out of 4217 DEMs, 2315 were upregulated and 1902 were downregulated (Figure 2(a)). There were 135 upregulated and 149 downregulated DELs (Figure 2(b)). 201 DEMis were upregulated, and the remaining 85 were downregulated (Figure 2(c)).

### 3.2. Construction of ceRNA Network

**Figure 3:**
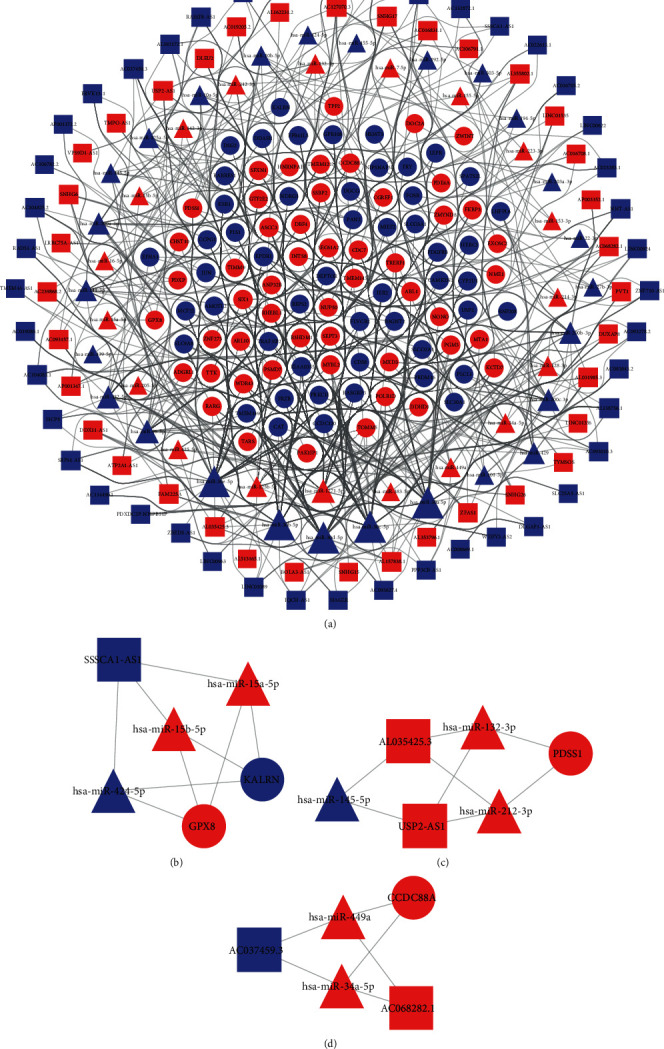
ceRNA regulation network (a) and three significant modules identified by MCODE in Cytoscape (b–d). Ellipse indicates mRNAs, triangle indicates miRNAs, and rectangle indicates lncRNAs. Red represents upregulated and blue represents downregulated.

Through GDCRNAtools, 103 DEMs, 80 DELs, and 45 DEMis were identified in the ceRNA network including 440 miRNA-mRNA and 418 lncRNA-miRNA regulatory associations or “edges”(Figure 3(a)). Through MCODE in Cytoscape, three significant modules were identified. The first module included two DEMs (KALRN and GPX8), three DEMis (hsa-miR-15a-5p, hsa-miR-15b-5p and hsa-miR-424-5p), and one DEL (SSSCA1-AS1) (Figure 3(b)). The second module consisted of one DEM (PDSS1), three DEMis (hsa-miR-145-5p, hsa-miR-212-3p and hsa-miR-132-3p), and two DELs (AL035425.3 and USP2-AS1) (Figure 3(c)). The third module consisted of one DEM (CCDC88A), two DEMis (hsa-miR-34a-5p and hsa-miR-449a), and two DELs (AC037459.3 and AC068282.1) (Figure 3(d)).

### 3.3. PPI Network Construction

**Figure 4:**
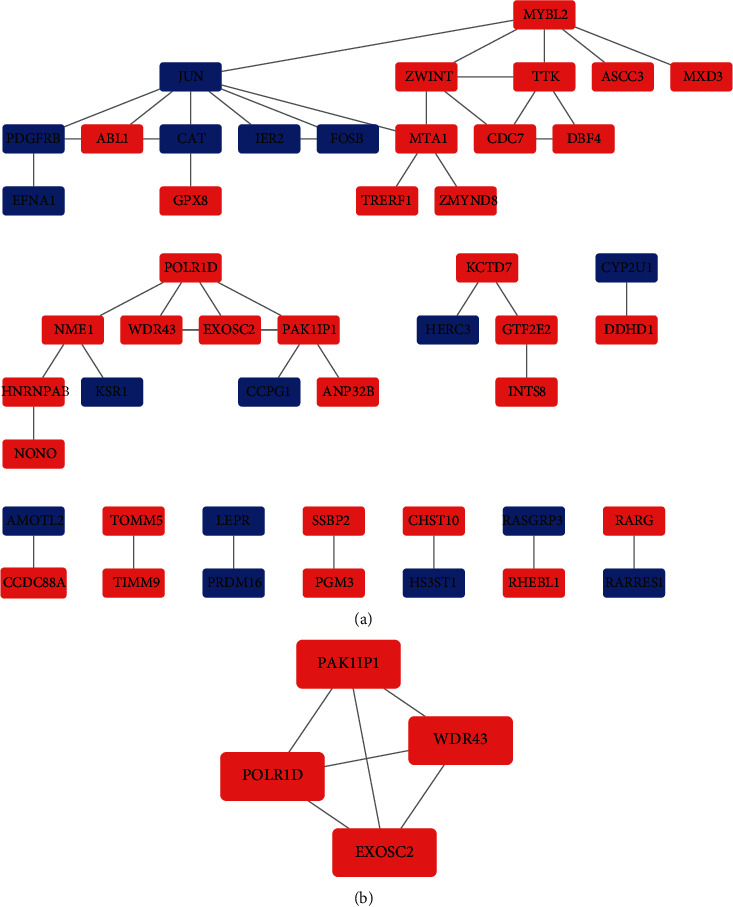
PPI network of target genes in the ceRNA network (a) and densely connected regions of the PPI network identified by MCODE (b). Red represents upregulated and blue represents downregulated.

The PPI network of the 103 DEMs including 52 upregulated and 51 downregulated involved in the ceRNA network was established (Figure 4(a)). Also following MCODE analysis, one hub-gene cluster consisting of four core genes (EXOSC2, PAK1IP1, WDR43, and POLR1D) was identified (Figure 4(b)). These four key genes, which were all upregulated interestingly, may play an essential role in RTK progression.

### 3.4. GO and KEGG Pathway Analyses

**Figure 5:**
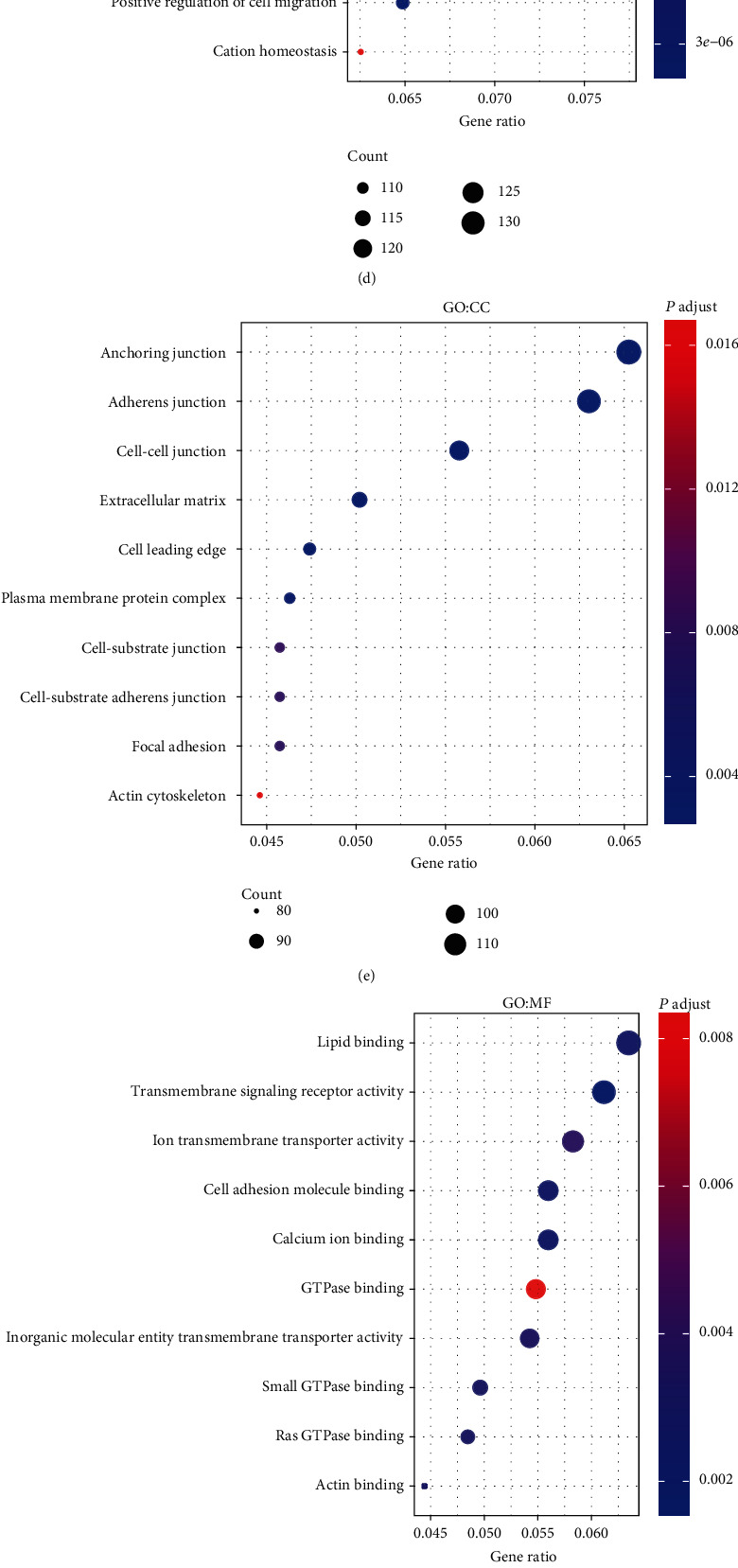
GO and KEGG pathways of up- and downregulated genes. (a–c) The bubble plots showing GO functional enrichment analysis for genes that were upregulated. (d–f) The bubble plots showing GO functional enrichment analysis for downregulated genes. (g–i) The bubble and bar plots showing KEGG pathways analysis of up- and downregulated genes. GO: Gene Ontology; BP: biological process; CC: cellular component; MF: molecular function; KEGG: Kyoto Encyclopedia of Genes and Genomes.

To explore the potential biological functions of the differentially expressed genes, GO and KEGG pathway enrichment analyses were conducted. In GO functional enrichment terms, BPs (356 up and 686 down), CCs (126 up and 56 down), and MFs (53 up and 81 down) were identified, respectively. The upregulated genes were significantly enriched in BPs (Figure 5(a)) including “ribonucleoprotein complex biogenesis”, “DNA repair”, and “establishment of protein localization to organelle”; CCs (Figure 5(b)) including “nuclear chromosome”, “nuclear chromosome part”, and “chromatin”; and MFs (Figure 5(c)) including “chromatin binding”, “structural constituent of ribosome”, and “catalytic activity, acting on RNA”. Correspondingly, the downregulated genes were significantly enriched in BPs (Figure 5(d)) including “blood vessel morphogenesis”, “angiogenesis”, and “inflammatory response”; CCs (Figure 5(e)) including “anchoring junction”, “adherens junction”, and “cell-cell junction”; and MFs (Figure 5(f)) including “lipid binding”, “transmembrane signaling receptor activity”, and “ion transmembrane transporter activity”. The KEGG pathway analyses showed the upregulated genes were significantly enriched in 14 pathways including “ribosome”, “RNA transport”, “cell cycle”, “spliceosome”, and “alcoholism”, while the downregulated genes were significantly enriched in 25 pathways including “pathways in cancer”, “PI3K-Akt signaling pathway”, “human papillomavirus infection”, “MAPK signaling pathway”, and “focal adhesion”(Figures 5(g)–5(i)).

### 3.5. Construction of Risk Scores Based on RNAs for Survival Analyses

**Figure 6:**
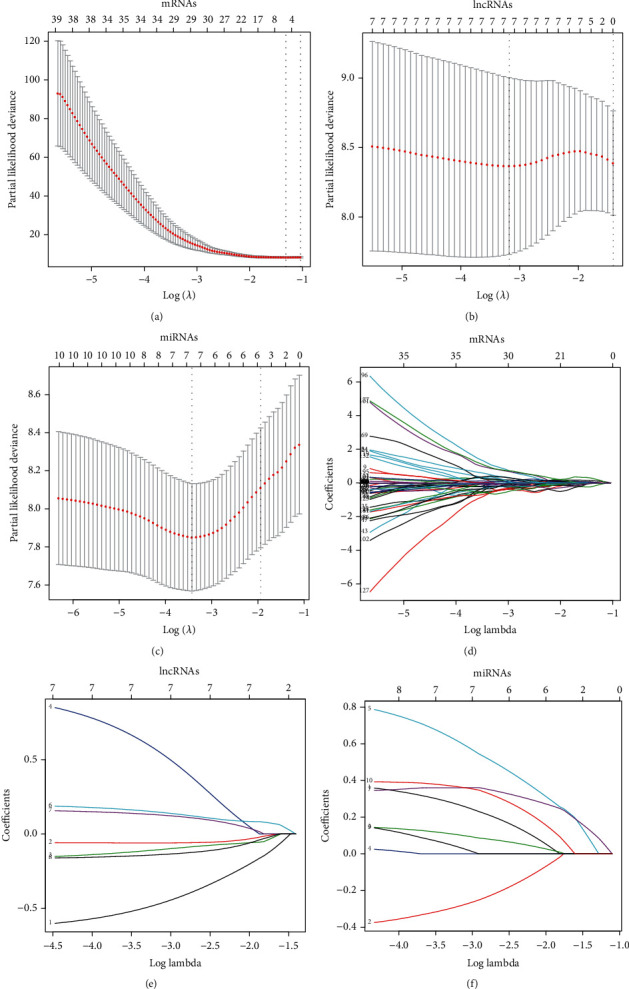
10-fold cross-validation and lambda min (a–c) and coefficient profile (d–f) of mRNAs, lncRNAs, and miRNAs in the LASSO model.

**Figure 7:**
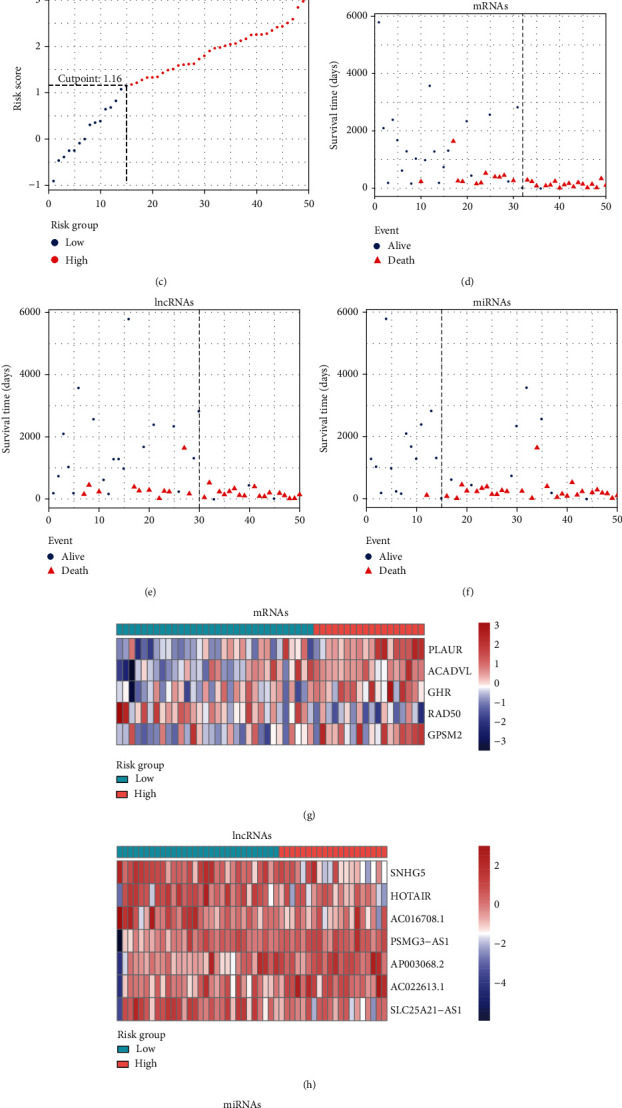
Risk score distribution (a–c), survival status (d–f), and the differentially expressed levels of identified mRNAs, lncRNAs, and miRNAs via heat map plot (g–i) for patients in low- and high-risk groups.

**Figure 8:**
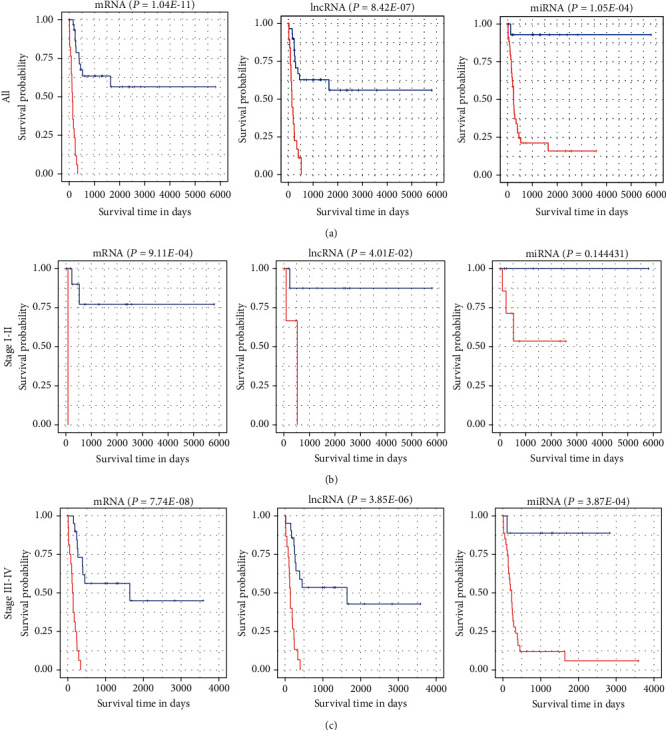
Kaplan-Meier survival analyses (a) and its subgroup analyses based on stages I-II (b) and III-IV (c) for patients in low- and high-risk groups represented by the blue and red curves, respectively.

Among the differentially expressed RNAs, 211 mRNAs, 8 lncRNAs, and 12 miRNAs associated with OS were obtained via the univariate Cox analysis. Then, 5 mRNAs (PLAUR, ACADVL, GHR, RAD50, and GPSM2), 7 lncRNAs (SNHG5, HOTAIR, AC016708.1, PSMG3-AS1, AP003068.2, AC022613.1, and SLC25A21-AS1), and 7 miRNAs (hsa-miR-22-5p, hsa-miR-199a-5p, hsa-miR-212-3p, hsa-miR-128-1-5p, hsa-miR-424-5p, hsa-miR-542-5p, and hsa-miR-769-3p) were identified using LASSO Cox regression model analysis (Figure 6). Moreover, the coefficients of identified RNAs signatures were calculated, and the formulas of the prognostic index model were imputed, respectively, as follows: (0.1148 × expression value of PLAUR) + (0.2591 × expression value of ACADVL) + (0.02925 × expression value of GHR) + (−0.1355 × expression value of RAD50) + (0.03125 × expression value of GPSM2) in mRNAs, (−0.4556 × expression value of SNHG5) + (−0.05992 × expression value of HOTAIR) + (−0.1043 × expression value of AC016708.1) + (0.5186 × expression value of PSMG3 − AS1) + (0.1395 × expression value of AP003068.2) + (0.1243 × expression value of AC022613.1) + (−0.1373 × expression value of SLC25A21 − AS1) in lncRNAs, and (0.2033 × expression value of hsa‐miR‐22‐5p) + (−0.3799 × expression value of hsa‐miR‐199a‐5p) + (0.1156 × expression value of hsa‐miR‐212‐3p) + (0.6258 × expression value of hsa‐miR‐128‐1‐5p) + (0.2997 × expression value of hsa‐miR‐424‐5p) + (0.02988 × expression value of hsa‐miR‐542‐5p) + (0.3347 × expression value of hsa‐miR‐769‐3p) in miRNAs. Also, the risk scores for RTK patient were calculated and all the patients were divided into high- and low- groups, respectively, based on the optimal cutoff values for risk scores (Figure 7, Table 2). Patients in each high-risk group had a significantly worse OS than those in the corresponding low-risk group (Figure 8(a), Table 3). Notably, the subgroup analyses based on stage revealed that the prognostic value of risk scores was independent of stage, except for miRNAs risk score in stage I-II subgroup (*P* = 0.1444)(Figures 8(b) and 8(c), Table 3).

### 3.6. Establishment and Validation of a Genomic-Clinicopathologic Nomogram

**Figure 9:**
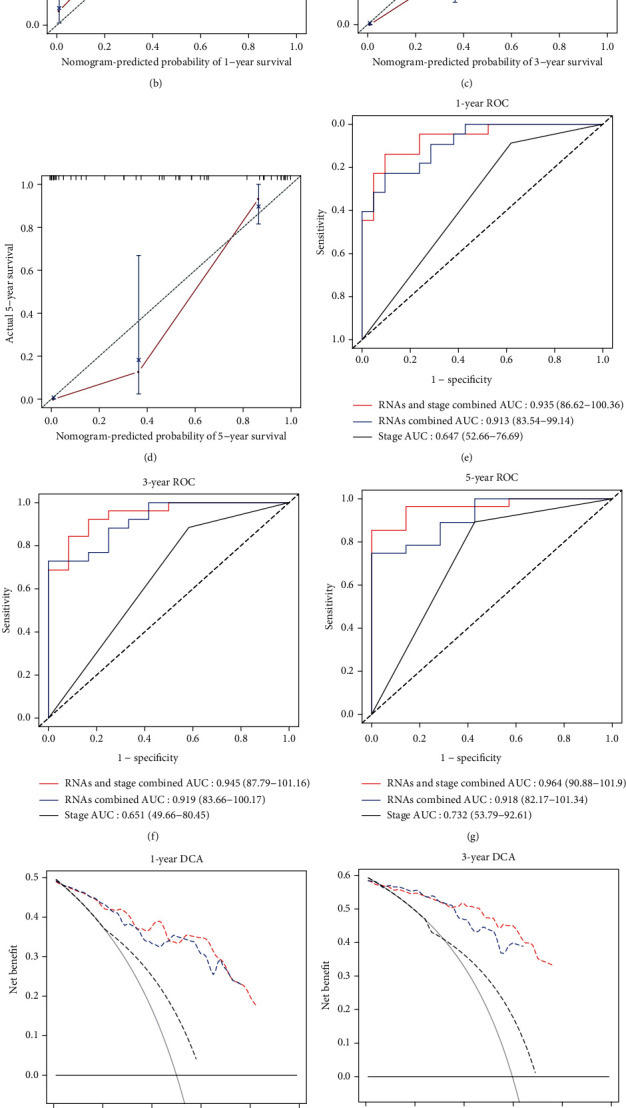
Establishment and validation of the predictive nomogram. The nomogram (a), the calibration curves (b–d), the time-dependent ROC curves (e–g), and the DCA curves (h–j) of the nomogram for predicting probabilities of patients with 1-, 3-, and 5-year OS. ROC: receiver operating characteristic; DCA: decision curve analysis.

To more accurately determine the prognostic value of risk scores based on RNAs (mRNAs, lncRNAs, and miRNAs), univariate and multivariate Cox regression analyses were performed. According to the results from univariate Cox analysis, the stage and risk scores were all significantly associated with OS for RTK patients (Table 4). The subsequent multivariate Cox analysis further demonstrated that the risk scores based on mRNAs and miRNAs remained powerful and independent prognostic factors (*P* = 0.01078 and *P* = 0.00816) (Table 4). Through integrating the stage and risk scores, a genomic-clinicopathologic predictive nomogram (combined model) was developed (Figure 9(a)). The calibration curves also showed high consistency between the actual proportion of 1-, 3-, and 5-year OS and the nomogram-predicted probability (Figures 9(b)–9(d)). Furthermore, the predictive accuracy of nomogram was evaluated by time-dependent ROC curves among three models including stage, RNAs combined, and RNAs and stage combined. The results illustrated the RNAs and stage combined model had a significantly greater AUC than that of the stage model (1-year, *P* = 1.05*e* − 6; 3-year, *P* = 5.17*e* − 5; 5-year *P* = 0.0117) (Figures 9(e)–9(g), Table 5, Table 6). Additionally, DCA curves illustrated that the net benefit for the combined model was higher than that for the stage model, verifying the clinical value of the genomic-clinicopathologic nomogram (Figures 9(h)–9(j)).

## 4. Discussion

RTK is a rare, highly aggressive type of cancer accounting for only 2% of all renal tumors in childhood [39]. There are only about 20 to 25 new cases of malignant rhabdoid tumor diagnosed each year in the United States. In Europe, only 107 RTK patients were identified from 1993 to 2005 [2]. The overall outcomes of RTK remain poor, although the prognosis for limited patients has improved partly [1, 3, 4]. Consequently, researchers keep on searching for the molecular pathogenesis of RTK and novel treatment strategies for improving prognosis in children with RTK. Even though previous studies have demonstrated that the majority of rhabdoid tumors arise as a consequence of homozygous inactivation of the SMARCB1 (INI1/SNF5/BAF47) gene [5], potential biomarkers to predict the prognostic outcomes in children with RTK have rarely been identified.

Lately, the essential roles of the ceRNA network in gene expression regulation have aroused interest of researchers. In the present study, we first identified differentially expressed mRNAs, lncRNAs, and miRNAs in patients with RTK. Then, 103 DEMs, 80 DELs, and 45 DEMis were selected to construct a ceRNA regulatory network, and three significant modules were revealed through cluster analyses. Subsequently, we constructed the PPI network of the 103 DEMs including 52 upregulated and 51 downregulated involved in the ceRNA network, and a hub-gene cluster consisting of four core genes (EXOSC2, PAK1IP1, WDR43, and POLR1D) was identified. GO and KEGG pathway enrichment analyses also revealed the functional characteristics of differentially expressed mRNAs, which demonstrated that the variations in biological processes, cellular components, molecular functions, and pathways may play an essential role in the pathogenic mechanism of RTK, necessitating further research to verify.

As far as we know, biomarker-based prognostic models or combined models built for predicting the outcomes of children with RTK have not been reported. In the present study, reasonable normalization of the raw data was achieved to eliminate the influence of differences in the platforms, prior to screening the prognosis-associated RNAs (mRNAs, lncRNAs, and miRNAs) for modeling. Moreover, the procedures of identifying the optimal RNAs for modeling were precise, such as the selection of the differentially expressed RNAs at step one, the selection through univariate Cox regression analysis at step two, and the selection through LASSO analysis at step three. Ultimately, 5 mRNAs, 7 lncRNAs, and 7 miRNAs were identified and the risk score formulas were constructed correspondingly. The following evaluations further confirmed the predictive value of risk scores for OS in children with RTK.

Nomograms, which are commonly used tools to estimate prognosis in oncology and medicine, can generate an individual probability of clinical events by incorporating multiple prognostic characteristics [40]. In the present study, a genomic-clinicopathologic nomogram was constructed by integrating the stage and risk scores based on RNAs. The combined model demonstrated significantly higher predictive accuracy than that of the stage model, which was further validated through calibration curves and time-dependent ROC curve analyses. The following DCA also verified the higher clinical value of the combined model compared with that of the stage model.

However, there were still several limitations to the present study. First of all, the sample size of RTK was small because RTK is a rare tumor. Second, for the same reason, neither an external validation cohort for the prognostic model nor the collection of RTK samples to verify the results of bioinformatics through RT-qPCR was available, and all the data we analyzed were collected from the GDC Data Portal, which might result in some bias. Finally, the results were not further validated in cell lines.

## 5. Conclusions

In summary, this study for the first time identified a ceRNA network that potentially regulated RTK progression, revealed probable genes and pathways associated with RTK, and constructed a genomic-clinicopathologic nomogram by integrating the stage and RNAs-based prognostic index, which might present a practicable tool for predicting the prognosis in children with RTK.

## Figures and Tables

**Table 1:** Clinicopathological characteristics of 50 RTK patients.

Characteristics	Median (range) or *n* (%)
Age at diagnosis in months	11.63 (0.10-179.53)
Gender	
Female	24 (48%)
Male	26 (52%)
Protocol	
NWTS-5	13 (26%)
AREN03B2	37 (74%)
Stage	
I-II	13 (26%)
III-IV	37 (74%)
Survival status	
Alive	22 (44%)
Dead	28 (56%)

NWTS-5: the fifth National Wilms' Tumor Study; AREN03B2: clinical trials cooperative group protocol name code.

**Table 2:** The cutoff values for risk scores of RNAs and the number of patients in different groups based on the cutoff values.

	mRNAs	lncRNAs	miRNAs
Cutoff values	1.663179	-2.15316	1.155198
Risk group			
Low	32	30	15
High	18	20	35

**Table 3:** The results of survival analyses and subgroup analyses based on the stage via log-rank test.

	mRNAs		lncRNAs		miRNAs	
	Chi-square value	*P* value	Chi-square value	*P* value	Chi-square value	*P* value
All	46.25	1.04*E* − 11	24.26	8.42*E* − 07	15.05	1.05*E* − 04
Subgroup						
Stages I-II	11.00	9.11*E* − 04	4.21	4.01*E* − 02	2.13	0.1444
Stages III-IV	28.87	7.74*E* − 08	21.34	3.85*E* − 06	12.60	3.87*E* − 04

**Table 4:** Univariate and multivariate Cox regression analyses of overall survival for RTK patients.

Risk factors	*N*	Univariate analysis			Multivariate analysis		
		HR	95% (CI)	*P* value	HR	95% (CI)	*P* value
Age	50	0.9995	0.9987-1	0.268			
Gender							
Female	24	1		0.233			
Male	26	0.6295	0.2942-1.347				
Protocol							
NWTS-5	13	1		0.522			
AREN03B2	37	0.7644	0.336-1.739				
Stage							
I-II	13	1		0.0248	1		0.11831
III-IV	37	3.9569	1.191-13.15		2.7683	0.7714-9.934	
Risk scores based on mRNAs	50	122.5950	23.41-642	1.25*E* − 08	17.4276	1.9371-156.792	0.01078
Risk scores based on lncRNAs	50	4.0815	2.227-7.481	5.36*E* − 06	1.8739	0.9245-3.798	0.08146
Risk scores based on miRNAs	50	3.6148	1.91-6.84	7.83*E* − 05	2.4325	1.2591-4.700	0.00816

HR: hazard ratio; CI: confidence interval; NWTS-5: the fifth National Wilms' Tumor Study; AREN03B2: clinical trials cooperative group protocol name code.

**Table 5:** 1-, 3-, and 5-year AUC (95% CI) among three models.

	Stage		RNAs combined		RNAs and stage combined	
	AUC	95% (CI)	AUC	95% (CI)	AUC	95% (CI)
1-year	0.6467	52.66-76.69	0.9134	83.54-99.14	0.9349	86.62-100.36
3-year	0.6506	49.66-80.45	0.9192	83.66-100.17	0.9447	87.79-101.16
5-year	0.7320	53.79-92.61	0.9175	82.17-101.34	0.9639	90.88-101.9

AUC: the area under the time-dependent receiver operating characteristic curve; CI: confidence interval.

**Table 6:** The results (*P* values) of comparing AUCs between any two models.

	RNAs and stage combined versus RNAs combined	RNAs and stage combined versus stage	RNAs combined versus stage
1-year	0.385975365	1.05*E* − 06	0.000406629
3-year	0.414081717	5.17*E* − 05	0.003882896
5-year	0.297225786	0.011765801	0.129916394

## Data Availability

The data used to support the findings of this study are included in the article.
